# Effects of Changes in Colored Light on Brain and Calf Muscle Blood Concentration and Oxygenation

**DOI:** 10.1100/tsw.2011.118

**Published:** 2011-06-09

**Authors:** J. Weinzirl, M. Wolf, P. Heusser, M. Nelle, U. Wolf

**Affiliations:** ^1^ Institute of Complementary Medicine KIKOM, University of Bern, Bern, Switzerland; ^2^ Biomedical Optics Research Laboratory, Division of Neonatology, University Hospital, Zurich, Switzerland; ^3^ Center for Integrative Medicine, University of Witten-Herdecke, Witten, Germany; ^4^ Division of Neonatology, University Hospital Bern, Bern, Switzerland

**Keywords:** color, color light therapy, near-infrared spectroscopy, NIRS, physiology, brain, muscle, hemodynamics, oxygenation functional study

## Abstract

Color light therapy is a therapeutic method in complementary medicine. In color therapy, light of two contrasting colors is often applied in a sequential order. The aim of this study was to investigate possible physiological effects, i.e., changes in the blood volume and oxygenation in the brain and calf muscle of healthy subjects who were exposed to red and blue light in sequential order. The hypothesis was that if a subject is first exposed to blue and then red light, the effect of the red light will be enhanced due to the contrastingly different characteristics of the two colors. The same was expected for blue light, if first exposing a subject to red and then to blue light. Twelve healthy volunteers (six male, six female) were measured twice on two different days by near-infrared spectroscopy during exposure to colored light. Two sequences of colored light were applied in a controlled, randomized, crossover design: first blue, then red, and vice versa. For the brain and muscle, the results showed no significant differences in blood volume and oxygenation between the two sequences, and a high interindividual physiological variability. Thus, the hypothesis had to be rejected. Comparing these data to results from a previous study, where subjects were exposed to blue and red light without sequential color changes, shows that the results of the current study appear to be similar to those of red light exposure. This may indicate that the exposure to red light was preponderant and thus effects of blue light were outweighed.

## INTRODUCTION

Light is essential for humankind. Although man has become fairly independent of Nature's day-and-night cycle, man still relies on light. Light influences human beings in many ways, on the physical, emotional, or spiritual-mental level. Light affects various physiological processes in man[1,2], such as the production of melatonin, a hormone essential for sleep induction, of cortisol, a hormone released under stress, and of vitamin D, which is important for bone metabolism. Light deprivation or constant darkness may lead, for example, to mood disorders. There is also some evidence that light and deterioration of the circadian rhythm may play a role in the risk of contracting diseases, such as cancer and metabolic and cardiovascular disorders, although the findings are still inconclusive (reviews[3,4,5,6,7]). Besides being a natural companion of man, light and artificial light are generally used for illumination and in medicine for diagnostic as well as therapeutic purposes. For example, for neonatal jaundice, a phenomenon occurring in neonates and preterm infants due to their immature liver function, blue light is applied[8,9,10]. Blue light[11,12,13,14,15,16] and red light[17,18] are also used in dermatology, and bright white light is used in psychiatry for the treatment of seasonal affective disorders and depression[19,20,21,22,23]. It is known that blue light is strongly absorbed by the skin and red light penetrates tissue relatively deeply.

Colored light is also used in complementary medicine; however, no uniform concept exists, and forms and applications are manifold. Since it would extend the scope of this study to report in much detail on the various forms of colored light therapy, only general information is given. Colored light therapy is either used as stand-alone or within complementary medicine systems, such as anthroposophic medicine. The application of colored light in complementary medicine mostly aims at improving patients' vitality and emotional and spiritual-mental well-being. Color light therapy is performed by either irradiating patients with colored light (direct application) or by facilitating patients to perceive colored light; for example, by projecting colored light onto a screen (indirect application). Although different concepts exist, it is common that the choice of the colors depends on the therapeutic goal; so-called warm colors (yellow, orange, and red) are applied to induce activating effects and so-called cool colors (cyan, blue, and violet) are applied to induce calm and relaxation. In practice, either single colors or combinations of colors are employed. When a combination is used, light of different colors is usually sequentially applied: green followed by red, or blue followed by red. While color light therapy has been used for many years, knowledge about possible effects of colored light and, in particular, its indirect application on physiological parameters is yet scarce. Therefore, the aim of our study was to investigate, using noninvasive near-infrared spectrophotometry (NIRS), the possible effects of blue and red light on blood volume and tissue oxygenation in the brain and calf muscle in healthy subjects.

We focused on blue and red light as they are the two main colors used in medical treatment and hold opposite features as mentioned above.

In a previous study, we investigated the effects of red and blue light severally[24]. Blue light exposure led to decreased oxygen consumption in the brain and the muscle, which may indicate a state of increased relaxation. Blue and red light had significantly different effects on tissue oxygen saturation ($StO_2$), both in the brain and muscle[24].

Since colored light is often applied in sequential order, the aim of this study was to investigate the effects of red and blue light in a sequential order and, in particular, during the color changes. The hypothesis was that if a subject is first exposed to blue and then to red light, the effect of the red light will be enhanced due to the contrastingly different characteristics of the two colors. The same was expected for first exposing a subject to red and then to blue light.

## MATERIAL AND METHODS

### Study Design

The study had a controlled, randomized (by computer), crossover design.

### Participants

Thirteen healthy volunteers (seven male, six female) were measured during exposure to colored light. One male subject had to be excluded since he fell asleep during the measurement. The remaining twelve subjects had a mean age of 30 years, with a range of 18 to 44 years, mean height of 174 (159 to 190) cm, and mean weight of 65 (52 to 88) kg. Written informed consent was obtained prior to each measurement from all subjects.

## Material and Methods

Light sources were white light bulbs (60W, OSRAM, Germany) incorporated in two arrays of 12 light bulbs each. Blue light filters were mounted in front of one-half of the light bulbs and red light filters (both filters, Lee, Germany) in front of the other half (Fig. 1B). An electric circuit was built that enabled switching on the light bulbs equipped with blue or red color filters separately.

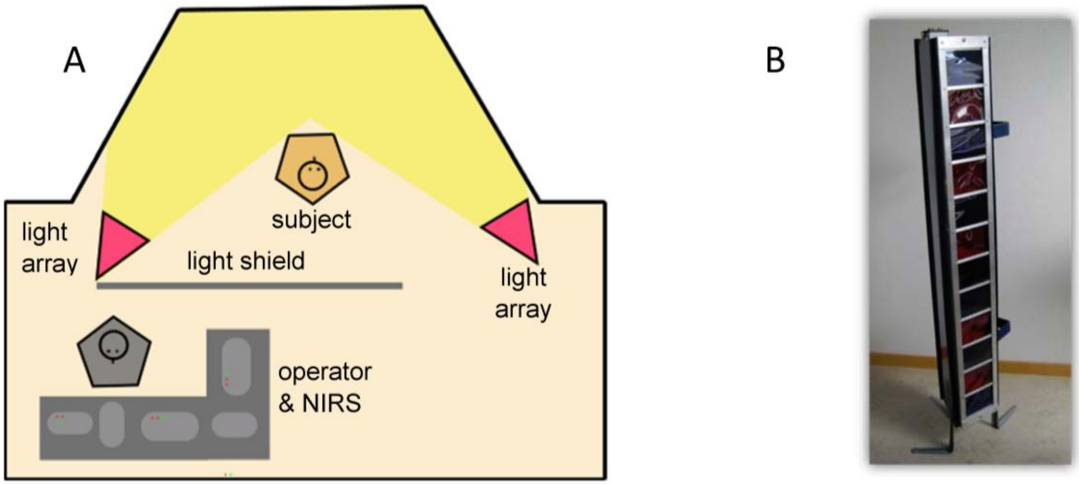

**FIGURE 1.** (A) Setup of the experiments. The subject was sitting in front of a white wall. During the nonexposure phases, the room was dark. During exposure, red or blue light was projected onto the wall by use of the light arrays, thereby resulting in an appearance of the wall in the respective color. The subject sat comfortably, with one near-infrared sensor attached to the left forehead and one sensor attached to the left calf muscle. The operator controlled the lighting and NIRS instrumentation. Light from the instrumentation was dimmed and additionally shielded. (B) Light exposure array. The figure shows one of two light bulb arrays, where blue and red light filters were alternatingly mounted to generate red and blue light for the exposure phases.

NIRS measurements were performed with a Hamamatsu NIRO 300 (Hamamatsu Photonics, Hamamatsu, Japan) continuous wave instrument. The instrument is based on the modified Lambert-Beer law and on spatially resolved near-infrared reflectance spectroscopy; operates at the wavelengths of 778, 813, 850, and 913 nm; and has two sensors. A more detailed description of the instrument can be found in Matcher et al.[25].

## Measurement Protocol

All subjects were measured twice on different days to avoid carryover effects. On the days of measurement, the subjects were exposed to the sequence of blue followed by red light or vice versa in a randomized, crossover protocol. Each subject was seated in a comfortable chair with armrests placed at a ~1.5-m distance from a white wall (Fig. 1A). In order to avoid artifacts, the subjects were instructed to move as little as possible and they were asked to keep their eyes open throughout the entire measurement.

One NIRS sensor was attached to the left forehead over the frontal lobe and at a location between Fp1 and F7 according to the international 10/20 system[26]. The second NIRS sensor was attached vertically on the left calf muscle, with the upper edge at a 10-cm distance from the fibula's head. The calf muscle was chosen because it is a well-studied muscle, has a sufficient muscle mass, and is less likely to be subject to movement artifacts than arm muscles. Both sensors were fixed with bandages. The protocol consisted of an 8-min baseline (darkness), 10-min exposure to colored light with either the sequence of blue/red or red/blue (5 min per color), followed by a 16-min recovery (darkness). During the exposure phases, colored light was projected onto the white wall, resulting in the wall appearing in the respective color. During the nonexposure phases, the room was completely dark and all instruments were shielded in order to avoid ambient light.

The protocol was approved by the Ethical Committee of the Canton of Basel, where the measurements were carried out due to logistic reasons and was in accordance with the Declaration of Helsinki.

## Measured Parameters

Relative concentration changes of oxyhemoglobin ($O_2Hb$ in µM), deoxyhemoglobin (HHb in µM), total hemoglobin (tHb in µM), redox state of cytochrome oxidase aa3 (Cyt in µM), absolute values of total hemoglobin concentration (THI in µM, also called tissue hemoglobin index by the manufacturer), and tissue oxygen saturation ($StO_2$ in %, also named tissue oxygenation index [TOI] by the manufacturer) were measured in the frontal lobe of the brain and in the calf muscle throughout the entire experiment.

While $O_2Hb$, HHb, tHb, and Cyt were calculated using the modified Lambert-Beer law[27], the calculation of THI and $StO_2$ was based on spatially resolved spectroscopy[25] according to the manufacturer of the NIRO 300 NIRS instrument. The Lambert-Beer law approach is sensitive to all tissue layers penetrated by the light and thus also to superficial tissue, such as skin and subcutaneous adipose tissue. The spatially resolved spectroscopy approach subtracts information from superficial tissue and is thus sensitive to deep layers of tissue[28], e.g., the brain. These two approaches enable us to obtain values from two different tissue regions, i.e., $O_2Hb$, HHb, tHb, and Cyt from superficial and deep tissue layers, and THI and $StO_2$ from deep tissue layers.

$StO_2$ represents the regional tissue oxygenation, and is mainly related to changes in the arterial oxygen saturation and blood flow, and only weakly to changes in mean arterial blood pressure and cerebral blood volume[29]. The relative tHb and THI represent changes or absolute values of hemoglobin concentration, and thus represent the blood volume of the tissue and are related to blood flow[30].

## Data Analysis

The concentrations of $O_2Hb$, HHb, tHb, Cyt, THI, and $StO_2$ were calculated by the software provided by the manufacturer (Hamamatsu Photonics, Hamamatsu, Japan). Data were manually screened for movement artifacts, which were removed. The first 3 min of the measurements were discarded because they may have contained initial transitions. The remaining 5 min of the baseline measurements were also tested for transition effects and since none were found, these 5 min constituted the initial baseline. Each measurement was divided into the following periods (5-min duration each): initial baseline, exposure to the first color, exposure to the second color, and three consecutive periods of recovery phase. For each of the periods, a median was calculated for each parameter and the values of the initial baseline were subtracted from the values of the other periods.

- *Temporal changes:* Using a paired t-test, the last 5-min period of the initial baseline was compared to the first and second period of the colored light exposure and to the three periods of recovery for each sequence (blue/red and red/blue) separately. In addition, the difference between the red and blue exposure for each sequence was analyzed by a t-test.
- *Comparison of the two color sequences:* Using a paired t-test, the values for the two sequences (blue/red and red/blue) were compared.
- *Other parameters:* By a linear mixed effects (LME) model (R statistical software), the influence of physiological parameters, such as age, weight, and height, on the measured parameters was tested.

## RESULTS

### Changes in Hemodynamics and Tissue Oxygenation in the Brain

- *Temporal changes:* The changes in the different parameters in the brain during and after exposure to blue/red and red/blue light are shown in Fig. 2. Although considerable changes were observed compared to the initial baseline, they were not significant due to a relatively large interindividual variability, except for the changes in the redox state of Cyt as indicated in Fig. 2. When the exposure switched from red to blue, a significant ($p$ = 0.027) decrease in the Cyt redox state occurred.
- *Comparison of the two color sequences:* Although the $O_2Hb$ and tHb traces appear different between the two sequences in Fig. 2., the physiological variability was high and, consequently, there was no statistically significant difference between the two sequences (blue/red and red/blue exposure) in the time evolution of the parameters during or after exposure, nor during the changes between colors.
- *Other parameters:* The analysis of the LME model to test for potential influence of age, weight, and height of the subjects on the results only yielded a significant negative influence of weight on the $StO_2$ ($p$ = 0.043).

### Changes in Hemodynamics and Tissue Oxygenation Saturation of the Calf Muscle

- *Temporal changes:* In the calf muscle, $O_2Hb$, tHb, THI, and, to a lesser extent, HHb concentration increased significantly during and after exposure for both sequences of blue/red and red/blue light (Fig. 3). When comparing the values during red to the values during blue, there were significant ($p$ < 0.01) differences in the values of $O_2Hb$, HHb, tHb, and THI for both the blue/red and red/blue exposures. This is likely to be due to the continuously growing blood pooling in the muscle (Fig. 3). In addition, $StO_2$ was significantly ($p$ = 0.013) different between the red and the blue condition for the red/blue exposure.
- *Comparison of the two color sequences:* There was no significant difference between the two sequences (blue/red and red/blue) in the time evolution of the parameters during or after exposure.
- *Other parameters:* The analysis of the LME model revealed a significant negative influence of age ($p$ = 0.004) and a positive influence of weight ($p$ = 0.005) on $O_2Hb$.

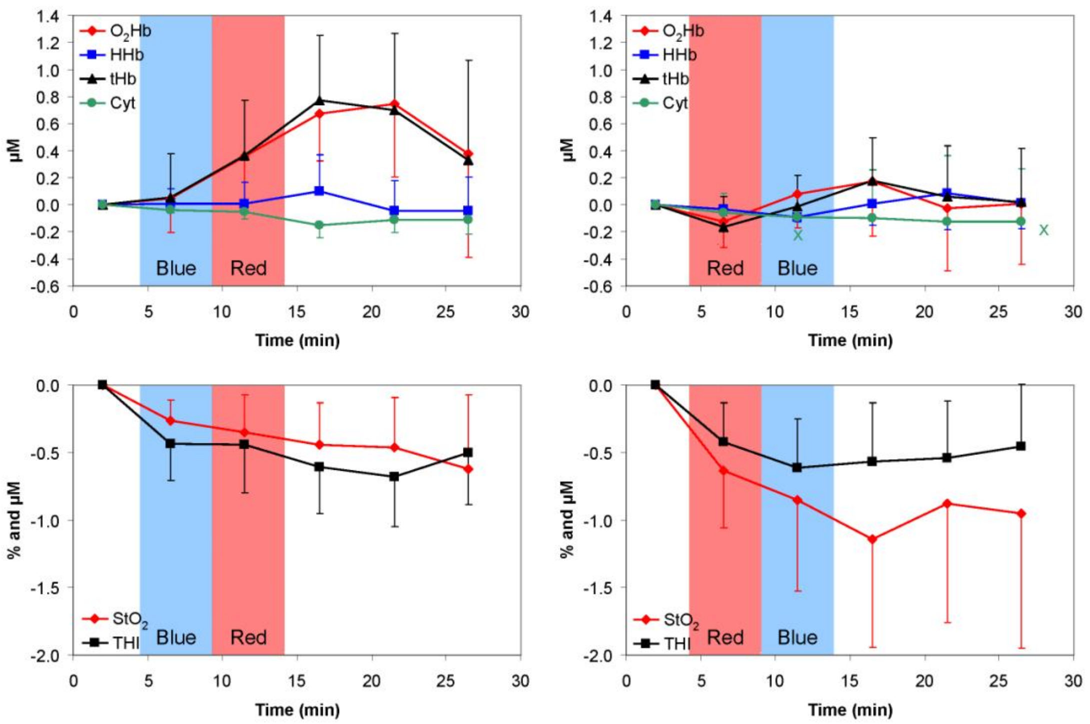

**FIGURE 2.** Brain: time evolution of the cerebral parameters. Left graphs: data from exposure to first blue and then to red light (shaded areas). Right graphs: data from exposure to first red then blue light (shaded areas). Upper graphs: NIRS parameters — $O_2Hb$ = oxyhemoglobin, HHb = deoxyhemoglobin, tHb = total hemoglobin, Cyt = cytochrome oxidase redox state. Lower graphs: NIRS parameters — $StO_2$ = tissue oxygen saturation, THI = total hemoglobin concentration. All parameters are shown relative to the baseline value before the exposure to colored light, which was set to zero. Two significant changes from baseline in Cyt are marked by an x ($p < 0.05$). Although there were considerably larger changes in some of the parameters, these changes were not significant. The values show a large interindividual variability (the whiskers indicate the standard error of mean).

## DISCUSSION

### Changes in the Brain

Although the $O_2Hb$ and tHb traces appear different between the two color sequences in Fig. 2, the physiological variability was high and, consequently, were not statistically significantly ($p$ = 0.13) different. The effect size, i.e., the mean difference of the effects, divided by the standard deviation corresponded to 0.37 for $O_2Hb$, which is generally considered to be close to a small effect size. The reason for this is a relatively large variation between subjects, which is represented by the large error bars (SEM) in Fig. 2. This variation originates from physiological changes and not from the NIRS instrument.

To interpret the observed changes, they are best compared to the previous experiments, which focused on exposure to light of single colors without sequential color changes[24]. In this previous study in the brain, the THI remained constant, which corresponds to an unchanged cerebral blood volume and blood flow. During exposure to blue light, a significant increase in $StO_2$ by 0.51% (maximum after exposure 0.98%) occurred, which corresponds to a decreased cerebral oxygen consumption, while during exposure to red light, $StO_2$ remained stable. There was a highly significant ($p$ = 0.001) difference in $StO_2$ during exposure to blue light compared to red light.

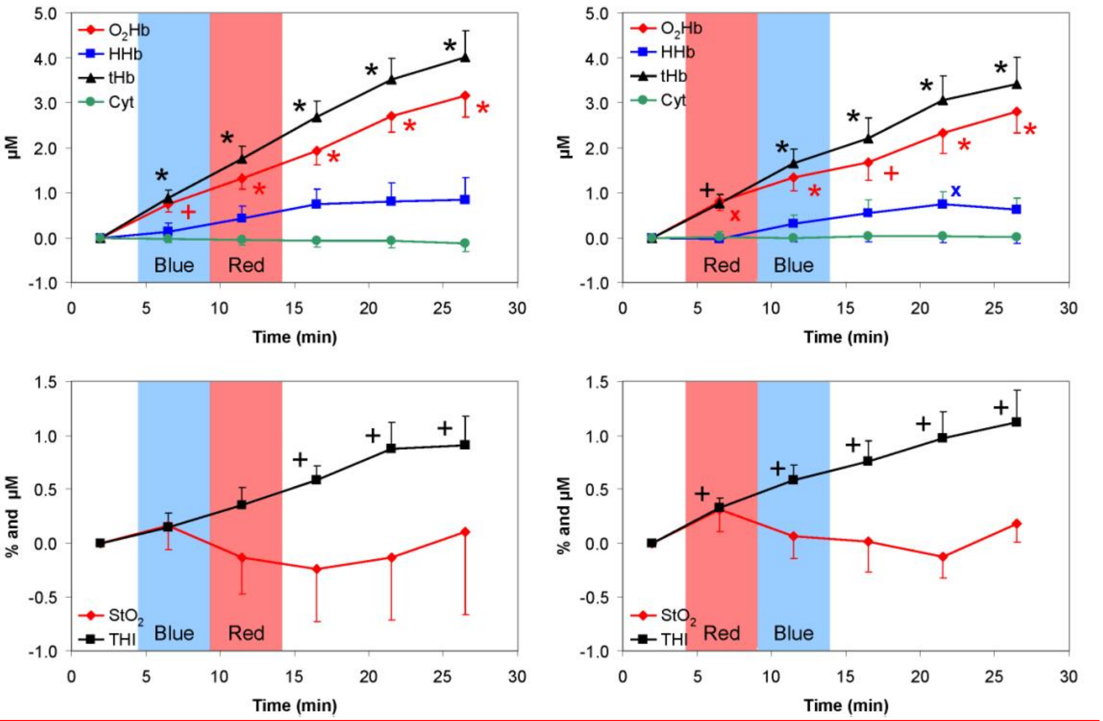

**FIGURE 3.** Calf muscle: time evolution of the muscle parameters. Left graphs: data from exposure to first blue and then to red light (shaded areas). Right graphs: data from exposure to first red then blue light (shaded areas). Upper graphs: NIRS parameters — $O_2$Hb = oxyhemoglobin, HHb = deoxyhemoglobin, tHb = total hemoglobin, Cyt = cytochrome oxidase redox state. Lower graphs: NIRS parameters — $StO_2$ = tissue oxygen saturation, THI = total hemoglobin concentration. All parameters are shown relative to the baseline value before the exposure to colored light, which was set to zero. Significant changes from baseline are marked by an x for $p < 0.05$, + for $p < 0.01$, and * for $p < 0.001$. There were considerably larger and highly significant increases in $O_2$Hb, tHb, and THI, which indicate blood pooling in the leg. The whiskers indicate the standard error of mean.

In the current study, there were no significant changes in any of the parameters (except Cyt) during and after the exposure, independent of the sequence (blue/red or red/blue). Thus, these changes are comparable to the changes in the previous study during red light exposure and different from the ones during blue light exposure. This could be interpreted as a preponderance of the effects excited by the red light over the effect of the blue light. This may be due to an outweighing effect of the red light or a too short exposure time (5 min) for the blue light to induce effects. Additionally, subjects were aware that after the blue light, the red light would appear. It is known from studies on the motor cortex that anticipating[31] or imagining[32] movements already induces changes in the cerebral blood volume and oxygenation. Thus, anticipation and imagery may have played a role in the current study.

The significant decrease in the redox state of Cyt (red/blue sequence only) may be interpreted as a decrease in oxygenation, oxygen consumption, or blood flow. Despite its significance, the change was very small.

It is noteworthy that tHb and THI seem to move in opposite directions even though the differences are not significant. This may indicate that the superficial tissue and the brain react in a differing way to the exposure to colored light.

Consequently, our hypothesis that the contrast between the two colors will enhance the effect of one of the colors is rejected. Nonetheless, the results indicate a predominating effect of the red light.

## Changes in the Calf Muscle

Again, for an easier understanding, we first summarize the findings of the previous publication[24]. In the leg, the THI significantly increased by 1.1 (blue) or 1.5 µM (red) continuously in the course of time and independently of the color, which was interpreted as reduced venous drainage, an accumulation of blood, and consequent venous pooling in the calf muscle during the measurement due to the sedentary position of the subjects. This effect was enhanced by sitting still, i.e., reducing the activity of the muscle pump. $StO_2$ remained relatively constant during the red light exposure measurements, but was increased during the blue light experiments, which was additionally, highly significantly ($p = 0.006$) different from the red light exposure. This corresponds to a decreased oxygen consumption excited by the blue light.

In the current study, the tHb, $O_2Hb$, and THI increased highly significantly for both sequences (blue/red and red/blue). Similar to the previous study, this can be explained by an accumulation of venous blood due to the sedentary position of the subjects during the measurement. The hypothesis of venous pooling is also supported by the slightly, although not significantly, decreased $StO_2$. The results of the current study are similar to the exposure to solely red light in the previous study, while the significant increase in $StO_2$ during exposure to solely blue light is absent. Again this may be due to an outweighing effect of the red light or a too short exposure time (5 min) for the blue light to induce an effect. In summary, it appears that the effects excited by the red light exposure are dominant and outweigh the effects of the blue light.

As for the brain, our hypothesis that the contrast between the two colors will enhance the effect of one of the colors is rejected. Nonetheless, the results suggest a preponderant effect of the red light.

## General Considerations, Limitations, and Outlook

The statistical significance of $p = 0.05$ means per definition that one out of 20 tests will lead to statistical significance by pure chance, which holds true for any study with this significance level. Thus, if, as in this study, several statistical tests are carried out, it has to be considered that in 5% of the tests, significances may be obtained just by chance. The LME model (R statistical software) already takes into account multiple testing and therefore corrects for it. For the paired t-test, five tests were carried per parameter. As usual for exploratory studies, we did not correct for multiple testing. For example, for the time evolution, a Bonferroni correction could be applied, which means that the significance level should be set at $p = 0.01$ instead of $p = 0.05$. In this study, a correction for multiple testing has no relevant effect on the interpretation of the results because few, but mostly high, significances were found

NIRS was used because it is completely noninvasive, does not use ionizing radiation or tracers, is compatible with other methods since it does not interfere electromagnetically (e.g., MRI, EEG)[33], is portable, is bedside usable, and is safe. Due to its advantages, NIRS has been used for many years in research and clinics, is ideally suited for functional studies[34], and is appreciated by subjects and staff. It yields continuous and quantitative measurements, and is highly sensitive, i.e., the instrumental noise is very low (our estimate for this study: ~0.01 µM). In addition, it enables studies in a natural environment, e.g., subjects in sedentary position, which is not possible using MRI or PET.

It would be worthwhile to include different aspects of exposure to colored light in future studies, e.g., different colors, intensities, durations, as well as other measurement locations and postures.

## CONCLUSION

No differences between the two sequences (blue/red vs. red/blue) and no enhancement of the effects of the second respective color were found. Surprisingly, also, no significant difference between the blue and red light was observed. The latter may seem in contrast to the previous study[24], where highly significant differences between red and blue were detected. In this study, the changes are similar to those observed for red light in the previous study.

In the case of the sequence red/blue, effects excited by the red light appear to persist during and after exposure to the blue light. This may be due to an outweighing effect of the red light or a too short exposure time (5 min) for the blue light to induce an effect.

In the case of the sequence blue/red, again the exposure to the blue light may have been too short to evoke a significant effect of the blue light before the red light appeared and, consecutively again, dominated the physiological effects.

In conclusion, it is most probable that the red light had preponderant effects that outweighed the effects of the blue light.

## ACKNOWLEDGMENT

We thank all subjects for their participation in this study. We thank Massimo Merlini (statistician, ETH Zurich) for statistical advice and valuable comments.

## References

1. Duffy, J.F. and Czeisler, C.A. (2009) Effect of light on human circadian physiology. *Sleep Med. Clin.* **4,** 165–177.
2. Morita, T. and Tokura, H. (1998) The influence of different wavelengths of light on human biological rhythms. *Appl. Hum. Sci.* **17,** 91–96.
3. Stevens, R.G. (2009) Working against our endogenous circadian clock: breast cancer and electric lighting in the modern world. *Mutat. Res.* **680,** 106–108.
4. Chung, S.A., Wolf, T.K., and Shapiro, C.M. (2009) Sleep and health consequences of shift work in women. *J. Womens Health (Larchmt.)* **18,** 965–977.
5. Mosendane, T. and Raal, F.J. (2008) Shift work and its effects on the cardiovascular system. *Cardiovasc. J. Afr.* **19,** 210–215.
6. Erren, T.C., Pape, H.G., Reiter, R.J., and Piekarski, C. (2008) Chronodisruption and cancer. *Naturwissenschaften* **95,** 367–382.
7. Costa, G., Haus, E., and Stevens, R. (2010) Shift work and cancer - considerations on rationale, mechanisms, and epidemiology. *Scand. J. Work Environ. Health* **36,** 163–179.
8. Maisels, M.J. and McDonagh, A.F. (2008) Phototherapy for neonatal jaundice. *N. Engl. J. Med.* **358,** 920–928.
9. Hansen, T.W. (2000) Pioneers in the scientific study of neonatal jaundice and kernicterus. *Pediatrics* **106,** E15.
10. Ip, S., Chung, M., Kulig, J., O'Brien, R., Sege, R., Glicken, S., Maisels, M.J., and Lau, J. (2004) An evidence-based review of important issues concerning neonatal hyperbilirubinemia. *Pediatrics* **114,** e130–153.
11. Hamilton, F.L., Car, J., Lyons, C., Car, M., Layton, A., and Majeed, A. (2009) Laser and other light therapies for the treatment of acne vulgaris: systematic review. *Br. J. Dermatol.* **160,** 1273–1285.
12. Shnitkind, E., Yaping, E., Geen, S., Shalita, A.R., and Lee, W.L. (2006) Anti-inflammatory properties of narrow-band blue light. *J. Drugs Dermatol.* **5,** 605–610.
13. Choudhary, S., Nouri, K., and Elsaie, M.L. (2009) Photodynamic therapy in dermatology: a review. *Lasers Med. Sci.* **24,** 971–980.
14. Buggiani, G., Troiano, M., Rossi, R., and Lotti, T. (2008) Photodynamic therapy: off-label and alternative use in dermatological practice. *Photodiagnosis Photodyn. Ther.* **5,** 134–138.
15. Brown, A.J., Fisher, C.H., and Kangas, K. (1993) Efficient, high-energy, narrow-band, blue-light source. *Opt. Lett.* **18,** 1177.
16. Noborio, R., Nishida, E., Kurokawa, M., and Morita, A. (2007) A new targeted blue light phototherapy for the treatment of acne. *Photodermatol. Photoimmunol. Photomed.* **23,** 32–34.
17. Darras-Vercambre, S., Carpentier, O., Vincent, P., Bonnevalle, A., and Thomas, P. (2006) Photodynamic action of red light for treatment of erythrasma: preliminary results. *Photodermatol. Photoimmunol. Photomed.* **22,** 153–156.
18. Zane, C., Capezzera, R., Pedretti, A., Facchinetti, E., and Calzavara-Pinton, P. (2008) Non-invasive diagnostic evaluation of phototherapeutic effects of red light phototherapy of acne vulgaris. *Photodermatol. Photoimmunol. Photomed.* **24,** 244–248.
19. Gross, F. and Gysin, F. (1996) [Phototherapy in psychiatry: clinical update and review of indications]. *Encephale* **22,** 143–148.

20. Radeljak, S., Zarkovic-Palijan, T., Kovacevic, D., and Kovac, M. (2008) Chromotherapy in the regulation of neurohormonal balance in human brain--complementary application in modern psychiatric treatment. *Coll. Antropol.* **32(Suppl 2),** 185–188.
21. Strong, R.E., Marchant, B.K., Reimherr, F.W., Williams, E., Soni, P., and Mestas, R. (2009) Narrow-band blue-light treatment of seasonal affective disorder in adults and the influence of additional nonseasonal symptoms. *Depress. Anxiety* **26,** 273–278.
22. Lieverse, R., Nielen, M.M., Veltman, D.J., Uitdehaag, B.M., van Someren, E.J., Smit, J.H., and Hoogendijk, W.J. (2008) Bright light in elderly subjects with nonseasonal major depressive disorder: a double blind randomised clinical trial using early morning bright blue light comparing dim red light treatment. *Trials* **9,** 48.
23. Even, C., Schroder, C.M., Friedman, S., and Rouillon, F. (2008) Efficacy of light therapy in nonseasonal depression: a systematic review. *J. Affect. Disord.* **108,** 11–23.
24. Weinzirl, J., Wolf, M., Nelle, M., Heusser, P., and Wolf, U. (2011) Colored light and brain and muscle oxygenation. *Adv. Exp. Med. Biol*., in press.
25. Matcher, S.J., Kirkpatrick, P., Nahid, K., Cope, M., and Delpy, D.T. (1995) Absolute quantification methods in tissue near infrared spectroscopy. In *Proceedings of Optical Tomography, Photon Migration, and Spectroscopy of Tissue and Model Media: Theory, Human Studies, and Instrumentation.* Pts 1 and 2. Vol. 2389. SPIE, Bellingham, WA. pp. 486–495.
26. Jasper, H.H. (1958) The ten-twenty electrode system of the International Federation. Electroencephalogr. *Clin. Neurophysiol.* **10,** 370–375.
27. Delpy, D.T., Cope, M., Vanderzee, P., Arridge, S., Wray, S., and Wyatt, J. (1988) Estimation of optical pathlength through tissue from direct time of flight measurement. *Phys. Med. Biol.* **33,** 1433–1442.
28. Franceschini, M.A., Fantini, S., Paunescu, L.A., Maier, J.S., and Gratton, E. (1998) Influence of a superficial layer in the quantitative spectroscopic study of strongly scattering media. *Appl. Optics* **37,** 7447–7458.
29. Tisdall, M.M., Taylor, C., Tachtsidis, I., Leung, T.S., Elwell, C.E., and Smith, M. (2009) The effect on cerebral tissue oxygenation index of changes in the concentrations of inspired oxygen and end-tidal carbon dioxide in healthy adult volunteers. *Anesth. Analg.* **109,** 906–913.
30. Grubb, R.L., Raichle, M.E., Eichling, J.O., and Ter-Pogossian, M.M. (1974) Effects of changes in Paco2 on cerebral blood volume, blood flow, and vascular mean transit time. *Stroke* **5,** 630–639.
31. Wolf, M., Wolf, U., Toronov, V., Michalos, A., Paunescu, L.A., Choi, J.H., and Gratton, E. (2002) Different time evolution of oxyhemoglobin and deoxyhemoglobin concentration changes in the visual and motor cortices during functional stimulation: a near-infrared spectroscopy study. *Neuroimage* **16,** 704–712.
32. Holper, L. and Wolf, M. (2010) Motor imagery in response to fake feedback measured by functional near-infrared spectroscopy. *Neuroimage* **50,** 190–197.
33. Toronov, V., Webb, A., Choi, J.H., Wolf, M., Michalos, A., Gratton, E., and Hueber, D. (2001) Investigation of human brain hemodynamics by simultaneous near-infrared spectroscopy and functional magnetic resonance imaging. *Med. Phys.* **28,** 521–527.
34. Wolf, M., Ferrari, M., and Quaresima, V. (2007) Progress of near-infrared spectroscopy and topography for brain and muscle clinical applications. *J. Biomed. Opt.* **12,** 062104.
